# Noise Resilience of Successor and Predecessor Feature Algorithms in One- and Two-Dimensional Environments

**DOI:** 10.3390/s25030979

**Published:** 2025-02-06

**Authors:** Hyunsu Lee

**Affiliations:** 1 Department of Physiology, School of Medicine, Pusan National University, Busandaehak-ro, Yangsan 50612, Republic of Korea; hyunsu.lee@pusan.ac.kr; 2 Research Institute for Convergence of Biomedical Science and Technology, Pusan National University Yangsan Hospital, Yangsan 50612, Republic of Korea

**Keywords:** reinforcement learning, successor features learning, predecessor feature learning, autonomous navigation, noisy environment

## Abstract

Noisy inputs pose significant challenges for reinforcement learning (RL) agents navigating real-world environments. While animals demonstrate robust spatial learning under dynamic conditions, the mechanisms underlying this resilience remain understudied in RL frameworks. This paper introduces a novel comparative analysis of predecessor feature (PF) and successor feature (SF) algorithms under controlled noise conditions, revealing several insights. Our key innovation lies in demonstrating that SF algorithms achieve superior noise resilience compared to traditional approaches, with cumulative rewards of 2216.88±3.83 (mean ± SEM), even under high noise conditions (σ=0.5) in one-dimensional environments, while Q learning achieves only 19.22±0.57. In two-dimensional environments, we discover an unprecedented nonlinear relationship between noise level and algorithm performance, with SF showing optimal performance at moderate noise levels (σ=0.25), achieving cumulative rewards of 2886.03±1.63 compared to 2798.16±3.54 for Q learning. The λ parameter in PF learning is a significant factor, with λ=0.7 consistently achieving higher λ values under most noise conditions. These findings bridge computational neuroscience and RL, offering practical insights for developing noise-resistant learning systems. Our results have direct applications in robotics, autonomous navigation, and sensor-based AI systems, particularly in environments with inherent observational uncertainty.

## 1. Introduction

Reinforcement learning (RL) has enabled significant progress in fields such as robotics and autonomous vehicles, with breakthrough technologies such as AlphaZero [1,2,3,4,5,6]. This computational framework describes the interaction between agents and their environment in optimizing reward signals [7]. The ultimate goal of RL is to discover the optimal policy, or the mapping between states and actions, that maximizes the expected cumulative reward. In this framework, agents learn to associate their actions with outcomes that lead to positive or negative rewards and use this association to optimize their policy. Beyond merely associating actions with rewards, advanced RL algorithms like AlphaZero demonstrate an unprecedented level of strategic depth and adaptability [1,2]. These agents not only learn the optimal policies linking states to actions for cumulative reward maximization but also exhibit innovative strategies and problem-solving skills. Advanced RL algorithms can equip agents with optimal policies for navigating not only virtual environments like games but also the diverse and unpredictable settings of the real world. For instance, RL enhances robotics with advanced human–robot collaboration [5] and aids autonomous vehicles in complex tasks like drone racing [3]. Additionally, RL efficiently enables robots to learn complex tasks like walking in a park, showcasing its effectiveness in varied and dynamic environments [6].

Advances in control systems have provided insights into managing the uncertainties and disturbances in learning environments. Traditional nonlinear control techniques have been used to develop methods to improve system stability and performance. State-filtered disturbance rejection control, for instance, employs state filters to handle both matched and unmatched disturbances, resulting in robust system performance without requiring consistently distinguishable disturbances [8]. This approach has proven effective in systems with irregular or unpredictable disturbance patterns. More recently, these capabilities have been further extended through integrating control systems with multilayer neural networks. Multilayer neural networks excel at approximating endogenous disturbances with high precision, offering new possibilities for adaptive control strategies [9]. However, even these sophisticated approaches face significant challenges when dealing with large exogenous disturbances, especially those of an incoherent kind. The complexity of control schemes in such situations highlights a persistent problem in the field: the trade-off between system robustness and implementability. The lessons from traditional control theory become especially important as RL moves from controlled environments to real-world applications.

Although RL has made significant progress, especially in controlled environments, there are significant challenges in applying it to real-world scenarios [10,11,12,13]. The inherent structure of RL methods is challenged by issues such as sample efficiency, scalability, generalization, and robustness. One of the most significant challenges for RL in real-world applications is its sensitivity to noisy observations, as real-world sensors invariably contain some level of noise [10,14,15]. Value-based approaches like Q learning [16] are vulnerable to noisy observations [17,18]. The issue of noise becomes more critical in the early stages of Q learning, as it can significantly amplify the errors and instability in Q-value estimates. This is problematic during initial exploration phases, where the agent is still learning to form policy decisions based on these values. In the early stages of learning, small sample sizes can cause biased estimates, leading to incorrect decision making. This can hinder the convergence of the learning process, necessitating a reset and relearning from a potentially better solution [17].

In contrast to the challenges faced by RL in real-world applications, animal intelligence exhibits remarkable efficiency and adaptability. In contrast to RL algorithms, which struggle with noisy data and require extensive training, animals demonstrate an innate ability to adapt quickly to changing environments. Both animals and humans require navigation and decision-making skills to locate resources, avoid dangers, and ultimately thrive in their environment. Mammals, birds, and reptiles use the hippocampus to encode spatial representations in order to organize and learn about relationships [19,20,21,22].

The discovery of place cells and grid cells in the hippocampus has led to the development of various hypotheses to explain how the brain processes spatial information [23,24,25,26,27]. One such theory is the predictive map theory proposed by Stachenfeld [28], which is based on the successor representation (SR) learning algorithm. The concept of SR learning includes the dissection of rewards and state transitions [29]. Consequently, SR learning involves representing the future expected occupancy of each state under a particular policy. This representation enables the agent to efficiently compute the expected value of all states given a policy without conducting an exhaustive search of the state space.

Recent studies have highlighted the advantages of the successor feature (SF) approach, a linear approximation extension of the SR algorithm, since it decouples the feature representation from the reward function and is thus suitable for knowledge transfer between domains. In the paper that first coined the term SF, the authors showed that SF facilitates the exchange of information across tasks and provides performance guarantees for the transferred policy even before learning begins [30]. Another notable advancement is the universal SF approximator, which combines the strengths of universal value function approximators, SF, and generalized policy improvement [31]. Furthermore, SF bridges model-based and model-free RL by predicting future observation frequencies and facilitating generalization across different inputs [32]. Together, these studies show the usefulness and versatility of SF in RL.

Despite extensive research on transfer learning employing SF [30,31,32,33], its robustness in noisy environments remains unexplored. While the predecessor feature (PF) approach, combining SF and eligibility trace, was proposed and shown to outperform SF [34,35], its robustness advantage in noisy environments has not been investigated. Initial investigations into SF and PF algorithms have shown promising results. A recent work Seo and Lee [36] examined these algorithms in a noisy T-maze environment, focusing on hyperparameter optimization. The study revealed that both the reward learning rate and eligibility trace decay parameters significantly influence adaptation performance under noisy conditions.

The experimental design in this study builds upon theoretical insights from neuroscience, particularly predictive map theory and SR learning. Given that SR learning aligns with hippocampal activity patterns in animal intelligence and recognizing that animals process state transitions and rewards separately during reward learning [37,38,39], we hypothesized that SF and PF algorithms would exhibit greater robustness to noise compared to value-based learning approaches such as Q learning or Q(λ) learning. However, the comparative performance of these biology-inspired approaches under noisy conditions remains understudied, particularly regarding the role of eligibility trace parameters in noise handling. This study addresses this gap by examining how SF and PF algorithms respond to varying noise levels, with a focus on their robustness compared to classical methods like Q learning and Q(λ) learning. To achieve this, experiments were conducted in grid worlds, where Gaussian noise was introduced into the observation vector.

Our study makes important contributions to our understanding of the performance degradation of RL algorithms in noisy environments by

- Providing the first empirical analysis comparing the robustness of SF and PF algorithms under varying levels of Gaussian noise.
- Demonstrating that, contrary to common assumptions, PF employing eligibility traces does not consistently outperform SF in noisy conditions.
- Revealing a nonlinear relationship between noise level and algorithm performance in two-dimensional (2D) environments, particularly influenced by the eligibility trace parameter (λ).
- Providing novel insights into optimizing the λ parameter for robustness to noise in both SF and PF implementations.

In this paper, we provide a comparative analysis of the SF and PF algorithms against Q learning and Q(λ) learning in environments with varying noise levels. Section 2.1 detailedly explains the *Markov decision processes* (MDPs) and delves into the mathematical and computational aspects of the SR learning and SF algorithms. Following this, Section 2.2 presents the implementation of the PF learning algorithm. Section 2.3 describes the establishment of noisy one-dimensional (1D) and 2D grid-world environments. Section 3 analyzes and contrasts the performance of RL algorithms—Q-learning, Q(λ)-learning, SF, and PF algorithms—in noisy environments. Finally, Section 4 discusses the theoretical underpinnings that potentially contribute to the robustness of the SF algorithm. This section also addresses the limitations of this study and elaborates on the broader implications of our findings, particularly in the realms of artificial intelligence (AI) and neuroscience research.

## 2. Materials and Methods

### 2.1. Background and Problem Formulation

#### 2.1.1. Markov Decision Processes

In this paper, we assume that RL agents interact with the environment through MDPs. The MDP used in this paper is a tuple M:=(S,A,R,γ) comprising the following elements: The set S is the states (i.e., information about the space), and the set A is the space of actions that the agent can take. A function R(s) maps the immediate reward received in state *s*. The discount factor γ∈[0,1) is a weight that reduces the value of future rewards.

The main task of the RL agent is to find a policy function that maximizes the total discounted reward, also known as the return $G_{t}=\sum_{i=t}^{\infty }\gamma^{i-t}R_{i+1}$, where $R_{t}=R\left(S_{t}\right)$. To solve this problem, we typically use *dynamic programming* methods to define and compute a value function $V^{\pi }\left(s\right):=E^{\pi }\left[G_{t}|S_{t}=s\right]$ that depends on policy π. The value function can be estimated using an approximation function $V^{w}\left(s\right)\approx V^{\pi }\left(s\right)$ parameterized by a weight vector $w\in R^{d}$. Here, R represents the real number space, and *d* represents the dimensionality of the state space, meaning w is a *d*-dimensional vector of real numbers. To update the weight vector, temporal difference (TD) learning can be utilized as follows: $w_{t+1}=w_{t}+\alpha \left[R_{t+1}+\gamma V^{w}\left(s_{t+1}\right)-V^{w}\left(s_{t}\right)\right]\nabla_{w}v_{w}\left(s_{t}\right)$. In this equation, α∈(0,1] serves as the learning rate parameter that controls the step size of weight updates, determining how much new information affects the current value estimates. The operator $\nabla_{w}$ denotes the gradient regarding the weight vector w, representing the direction of the steepest increase in the value function. The term $\left[R_{t+1}+\gamma V^{w}\left(s_{t+1}\right)-V^{w}\left(s_{t}\right)\right]$ represents the TD error, measuring the difference between the predicted value and the improved estimate. Here, $V^{w}\left(s_{t}\right)$ gives the current value estimate for state $s_{t}$, $R_{t+1}$ represents the immediate reward received, and $\gamma V^{w}\left(s_{t+1}\right)$ provides the discounted estimate of the future value. This updated equation adjusts the weights to minimize the difference between the predicted and observed values, with the learning rate α controlling the magnitude of these adjustments.

TD (0) refers to an algorithm that uses the typical one-step TD update rule as mentioned above, while TD (λ) refers to a classic algorithm that uses an eligibility trace based on past experience. The update rule for TD (λ) is defined as $w_{t+1}=w_{t}+\alpha \delta_{t}e_{t}$, where $\delta_{t}=R_{t+1}+\gamma V^{w}\left(s_{t+1}\right)-V^{w}\left(s_{t}\right)$ is referred to TD error and $e_{t}=\gamma \lambda e_{t-1}+\nabla_{w}V^{w}\left(s_{t}\right)$ is referred to the eligibility trace, where λ indicates the trace decay parameter.

#### 2.1.2. Successor Features Learning

The core idea of SR learning is that the value function can be decomposed into the expected visiting occupancy and reward of successor state $s^{\prime }$ as follows: $V^{\pi }\left(s\right)=\sum_{s^{\prime }}E^{\pi }\left[\sum_{i=t}^{\infty }\gamma^{i-t}I\left(S_{i}=s^{\prime }\right)R\left(s^{\prime }\right)|S_{t}=s\right]=\sum_{s^{\prime }}M\left(s,s^{\prime }\right)R\left(s^{\prime }\right)$, where $I(S_{i}=s^{\prime })$ returns 1 if the agent visits the successor state $s^{\prime }$ at time *t* and 0 otherwise. Thus, M(s,:) represents the discounted expectation of a visit from state *s* to its successor states, which can be called a successor state vector or SF.

Similar to how we use a weight vector w to estimate the value function, we can use a weight matrix and vector to estimate M(s,:) and $R(s^{\prime })$, respectively. In the tabular environment $R^{\left|S\right|}$, we can represent the state vector as a simple one-hot vector ϕ(s). Thus, we can factorize the reward vector as $R\left(s\right)=\phi \left(s\right)\cdot w^{r}$ and the SF as $M\left(s,:\right)=W^{sf}\phi \left(s\right):=\psi^{W}\left(s\right)$. Here, $\psi^{W}\left(s\right)$ represents the SFs for state *s*, computed using weight matrix W, capturing the expected future occurrences of features under the current policy. Similarly, $\psi^{\pi }\left(s\right)$ denotes the true SFs under policy π, representing the ideal prediction of the future state features that would be obtained with perfect knowledge of the environment’s dynamics. Therefore, we can rewrite the value function under policy π as $V^{\pi }\left(s\right)=\psi^{\pi }\left(s\right)\cdot w^{r}$ and the value approximation function as $V^{W}\left(s\right)=\psi^{W}\left(s\right)\cdot w^{r}$.

Using the TD update rules, we can update $w^{r}$ and $W^{sr}$ as follows:

(1) $$w_{t+1}^{r}=w_{t}^{r}+\alpha_{r}\left(R_{t}-\phi \left(s\right)\cdot w_{t}^{r}\right)\phi \left(s\right)$$

(2) $$W_{t+1}^{sf}=W_{t}^{sf}+\alpha_{W}\left[\phi \left(s_{t}\right)+\gamma \psi^{W}\left(s_{t+1}\right)-\psi^{W}\left(s_{t}\right)\right]⊗\phi \left(s_{t}\right)$$

These update equations follow from the original formulation of SR learning [29], which was later extended by Barreto et al. [30] for SF. Therefore, we present an example algorithm of SF in Algorithm 1.
**Algorithm 1** Successor feature learning.  1:**procedure** PF ($episodes,W^{sf},w^{r},\alpha_{W},\alpha_{r}$)  2:     initialize $W^{sf},w^{r}$  3:     **for** episode in 1…*n* **do**  4:        $s_{t}\leftarrow$ initial state of episode  5:        **for** pair ($s_{t},s_{t+1}$) and reward *R* in episode **do**  6:            $\delta^{sf}\leftarrow \phi \left(s_{t}\right)+\gamma \psi^{W}\left(s_{t+1}\right)-\psi^{W}\left(s_{t}\right)$  7:            $\delta^{r}\leftarrow R-\phi \left(s_{t+1}\right)\cdot w^{r}$  8:            $W^{sf}\leftarrow W^{sf}+\alpha_{W}\delta^{sf}$  9:            $w^{r}\leftarrow w^{r}+\alpha_{r}\delta^{r}\phi \left(s_{t+1}\right)$10:    **return** $W^{sf},w^{r}$

### 2.2. Predecessor Feature Learning

Similar to how the TD (0) algorithm can be converted into TD (λ) by using the eligibility trace, incorporating SF with the eligibility trace can result in the production of PF. The eligibility trace can be updated using the one-hot state vector ϕ(s) as follows: $e_{t}=\gamma \lambda e_{t-1}+\phi \left(s_{t}\right)$. Following the PF framework introduced by Bailey and Mattar [34] and Pitis [35], the weight matrix of the PF update rule can be written as follows:

(3) $$W_{t+1}^{pf}=W_{t}^{pf}+\alpha_{W}\left[\phi \left(s_{t}\right)+\gamma \psi^{W}\left(s_{t+1}\right)-\psi^{W}\left(s_{t}\right)\right]⊗e_{t}$$

where we use a linear approximation, $\psi^{W}\left(s\right)=W^{pf}\phi \left(s\right)$, for simplicity. By using a one-hot state feature vector, we consider PF and predecessor representation (PR) learning to be highly analogous. Therefore, we present an example of PF algorithms in Algorithm 2.
**Algorithm 2** Predecessor feature learning.  1:**procedure** PF ($episodes,W^{pf},w^{r},\lambda ,\alpha_{W},\alpha_{r}$)  2:     initialize $W^{pf},w^{r}$  3:     **for** episode in 1…*n* **do**  4:        $s_{t}\leftarrow$ initial state of episode  5:        e←0 (eligibility trace reset)  6:        **for** pair ($s_{t},s_{t+1}$) and reward *R* in episode **do**  7:            $e\leftarrow e+\phi (s_{t})$  8:            $\delta^{pf}\leftarrow \phi \left(s_{t}\right)+\gamma \psi^{W}\left(s_{t+1}\right)-\psi^{W}\left(s_{t}\right)$  9:            $\delta^{r}\leftarrow R-\phi \left(s_{t+1}\right)\cdot w^{r}$10:            $W^{pf}\leftarrow W^{pf}+\alpha_{W}\delta^{pf}⊗e$11:            $w^{r}\leftarrow w^{r}+\alpha_{r}\delta^{r}\phi \left(s_{t+1}\right)$12:            e←γλe13:    **return** $W^{pf},w^{r}$

Pitis [35] introduced the concept of “source traces” as an application of eligibility traces in SR learning. They showed the convergence of a TD (λ)-like source learning algorithm and developed a novel algorithm for learning the source (SR) map, which outperformed previous approaches. Bailey and Mattar [34] also proposed an algorithm based on the same idea as proposed in this paper, naming it “predecessor features”. The PF algorithm was applied to tabular and feature representations and outperformed the “ExpectedTrace” [40] algorithm on the Cartpole task. However, it has not been compared to the SF algorithm in a noisy environment.

### 2.3. Experimental Design

#### 2.3.1. Environments with Noise

**Figure 1:**
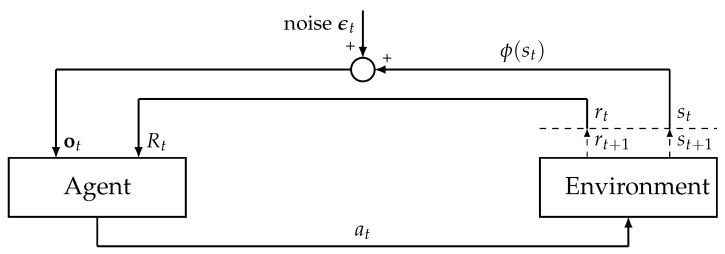
Schematic representation of environment–agent interactions in RL with noisy observations. The agent receives an observation $o_{t}$ and a reward $R_{t}$ at each time step *t*, based on which it decides on an action $a_{t}$. The environment processes this action to update its state to $s_{t+1}$, providing the next state and reward ($r_{t+1}$) to the agent. A noise term $\epsilon_{t}$ is added to the state representation $\phi (s_{t})$ before being fed back to the agent.

This section presents the noisy environment configuration adopted in our research. In our study, we considered a scenario within an MDP, where the state is denoted by a one-hot vector $\phi (s_{t})$, indicative of the agents’ positions within a grid world. We introduced a noisy MDP by adding a noise vector $\epsilon_{t}$ to $\phi (s_{t})$, simulating the effect of uncertainty in the agents’ perceived positions (Figure 1).

Following the approach recommended by [12,15], our study incorporated noise solely into the state observations while maintaining the state transition dynamics in the environment unchanged. This approach allowed the noisy environment to more accurately reflect measurement errors, including sensor errors. To account for the effects of sensor noise commonly encountered in real-world scenarios, we added a Gaussian noise term to the observation vector $o_{t}$ received by the agent in each state as follows:

(4) $$o_{t}=\phi \left(s_{t}\right)+\epsilon_{t},\;\epsilon_{t}∼N\left(0,\sigma^{2}I\right)$$

where $\epsilon_{t}$ is the Gaussian noise vector with zero mean and covariance matrix $\sigma^{2}I$, where I is the identity matrix. Since σ regulates the level of noise in $o_{t}$ as σ rises, $o_{t}$ becomes noisier. Equation (4) has been widely adopted in analyzing the impact of environmental noise on RL systems [12,15].

To evaluate the robustness of agents against a noisy observation $o_{t}$, we conducted experiments in both a simple 1D grid world and a more complex 2D grid world. The observation vector’s noise levels σ were set at [0.05, 0.25, 0.5] for low, medium, and high Gaussian noise to test the robustness of the agents. These levels were chosen to assess the algorithms’ performance across a spectrum of environmental challenges, ensuring a comprehensive evaluation of their resilience to different degrees of stochastic disturbances. In our investigation, the discount factor γ was set to 0.95.

**Figure 2:**
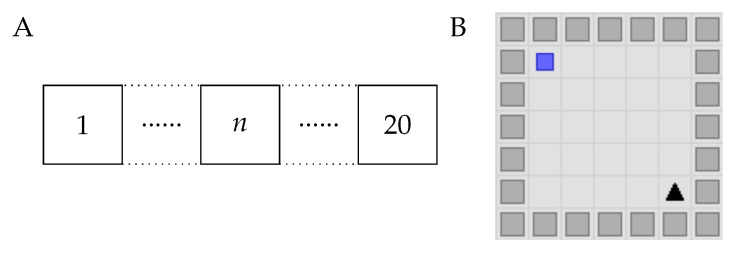
Schematic of the grid world following the MDP. (**A**) A 1D grid world comprising 20 states, labeled from 1 to 20. The agent begins at state 1 (leftmost), and the goal is to reach state 20 (rightmost). (**B**) A 2D grid world, structured as a 7 × 7 grid with barriers along the edges, restricting the agent’s movement to a 5 × 5 area. The starting position is marked in the bottom right corner (triangle), and the goal is located in the top left corner (square).

The 1D grid world in this study was modeled after the design used in our previous study [41]. It comprised 20 states, where the agent started at state 1 and aimed to reach state 20 (Figure 2A). When the agent successfully reached the target, it was awarded a reward of 1. If the target was not reached, the reward was 0. The agent’s action space comprised two actions: move right or move left.

Our 2D grid world was developed using the neuro-nav library [42], featuring a 7×7 grid with barriers along the edges, resulting in a 5×5 navigable space (Figure 2B). This design limited the agent’s movable states to 25. However, the 2D grid presented 49 observable states, adding complexity to the agent’s task because of the increased number of potential states to observe and interpret. In this 2D grid world, the agent’s aim was to navigate from the starting point in the bottom right corner to the goal located in the top left corner. Upon successfully reaching the target, the agent was awarded a reward of 1; otherwise, the award was 0. The agent’s action space comprised four actions: move up, down, right, or left.

#### 2.3.2. Algorithm Hyperparameters

Using our method, we evaluated the performance of four RL algorithms—Q learning, Q(λ) learning, SF, and PF—in a noisy environment.

Q learning, a foundational model-free RL algorithm [16], estimates action values through TD learning by using an update rule:

(5) $$Q\left(s,a\right)=Q\left(s_{t},a_{t}\right)+\alpha \left[R_{t+1}+\gamma \max\left(Q\left(s_{t+1},a\right)\right)-Q\left(s_{t},a_{t}\right)\right]$$

where α is the learning rate, γ is the discount factor, and $\max(Q\left(s_{t+1},a\right))$ represents the maximum Q value across all actions in the next state. Q(λ) learning extends this approach by incorporating eligibility traces, allowing the algorithm to propagate rewards to previously visited state–action pairs. This extension helps accelerate learning by updating multiple state–action pairs in each step, potentially providing better credit assignment in noisy environments.

Q learning and Q(λ) learning were selected because of benchmarking, providing a basis for comparison with SF and PF learning. For Q(λ) learning and PF, we varied the λ parameter at values of 0.7, 0.8, and 0.9 to determine the effect on the robustness of the algorithm under different levels of noise.

For the agent’s policy function in our experiments, an ϵ-greedy policy was implemented. This policy alternates between exploring random actions with a probability of ϵ and exploiting known actions with the highest Q-value estimate with a probability of 1−ϵ. To balance exploration and exploitation, the ϵ probability decays over episodes according to $\epsilon_{k+1}:=\max\left(0.99\cdot \epsilon_{k},0.01\right)$, where $\epsilon_{1}=1.0$, and *k* denotes the episode index.

For both Q learning and Q(λ) learning, the learning rate was set to 0.1. Similarly, for SF and PF learning, the feature weight learning rate $\alpha_{W}$ and the reward vector learning rate $\alpha_{r}$ were both assigned a value of 0.1.

#### 2.3.3. Evaluation Metrics

We implemented a comprehensive evaluation framework to ensure fair and rigorous comparison of the algorithms under noisy conditions. The experimental design comprised 100 independent runs, each comprising 3000 episodes, providing statistical robustness while accounting for the stochastic nature of RL algorithms.

For each algorithm, we standardized the testing conditions by setting consistent episode termination criteria: the episodes concluded either upon reaching the goal state or upon reaching the maximum step limit (100 steps for 1D grid world, 200 steps for 2D grid world). These limits were calibrated based on preliminary studies to allow sufficient exploration while preventing infinite loops in challenging noise conditions.

For the performance evaluation, we used two main metrics: cumulative reward and episode length. To mitigate the impact of short-term fluctuations and provide more stable trend analysis, we applied a moving average with a 20-episode window to the episode length trajectories. This window size was selected to balance noise reduction and the preservation of meaningful temporal patterns.

To ensure comprehensive statistical analysis and fair comparison of the algorithms, we computed multiple complementary metrics:

1. Central tendency measures:
  - Mean performance to assess average behavior;
  - Median performance to evaluate typical outcomes;
  - The standard error of the mean (SEM) to quantify estimation uncertainty.
2. Distribution characteristics:
  - The 25th and 75th percentiles to understand performance spread;
  - Mean/SEM ratio as a standardized metric of algorithm stability and consistency.

The mean/SEM ratio served as a crucial metric for fair comparison, as it normalizes performance by its variability, providing a dimensionless measure of algorithm reliability across the different noise conditions. This comprehensive statistical framework enabled objective comparison while accounting for the inherent stochasticity in RL algorithms.

## 3. Experimental Results

### 3.1. Comparison of RL Agents in a Noisy 1D Environment

In this section, we present the results of our study comparing the performance of the RL agents in a noisy 1D grid world. Our investigation into the robustness of the various RL agents under different noise conditions revealed notable distinctions in performance, particularly for SF. The experimental environment comprised a 1D grid world with 20 discrete states (Figure 2A), building upon our previous work [41]. We evaluated algorithm performance under three noise conditions (σ= 0.05, 0.25, and 0.50) to understand how the different approaches maintain their navigation efficiency as environmental uncertainty increases.

#### 3.1.1. Robustness and Consistency of SF Learning in Cumulative Reward Analysis

**Figure 3:**
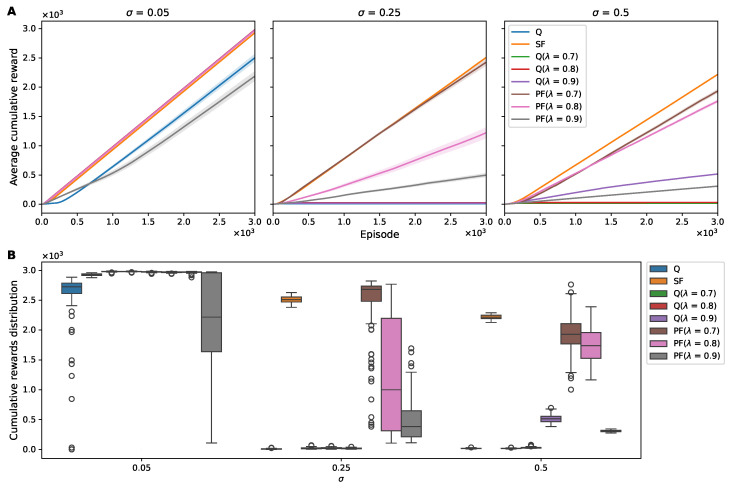
Cumulative reward of RL agents in a noisy 1D environment. (**A**) Average cumulative rewards over 3000 episodes for Q learning, Q(λ) learning, SF, and PF under different levels of observation noise (σ = 0.05, 0.25, 0.5). Each line represents the mean cumulative reward across episodes, with the shaded area depicting the SEM. (**B**) Distribution of cumulative rewards for each agent under various noise settings. Box plots illustrate the median (central line), interquartile range (box limits), and outliers (individual points) of the cumulative rewards obtained over 3000 episodes, providing a comparative view of the reward distributions and the robustness of each algorithm to varying noise intensities.

To evaluate how the different RL algorithms maintained performance under environmental noise, we first analyzed their cumulative reward patterns. Figure 3 presents a systematic analysis of the RL algorithm performance under the varying noise conditions in our 1D grid-world environment. The visualizations show how the different algorithms responded to increasing levels of environmental noise (σ = 0.05, 0.25, and 0.5), revealing both the temporal progression of learning and the final performance distributions.

Our analysis revealed distinct patterns in how the algorithms adapted to noise. In the 1D grid world with low Gaussian noise (σ = 0.05), all agents exhibited relatively high mean cumulative rewards, with Q(λ) learning demonstrating the least variability, as evidenced by the high mean/SEM ratios. However, the robustness of the SF agent became evident at increased noise levels (σ = 0.25 and 0.50). While other algorithms showed degraded performance under higher noise, the SF agent maintained a stable performance curve (Figure 3). This stability is further supported by the narrow interquartile ranges in Table 1.

At a noise level of σ = 0.25, while PF at λ = 0.7 exhibited a higher median cumulative reward than SF, the latter’s tighter interquartile spread translated into a greater mean value and a higher mean/SEM ratio (Figure 3B and Table 1). In the 1D grid world with high Gaussian noise (σ = 0.50), the SF algorithm not only preserved its performance, but it also degraded less than the other algorithms. This robustness was noteworthy in the mean/SEM ratio, which remained significantly higher for SF at σ = 0.50 compared to those of the Q-learning and PF algorithms (Figure 3B and Table 1). This metric emphasizes the robustness of SF in maintaining reward consistency under challenging noise conditions.

In contrast, the performance of the Q-learning, Q(λ)-learning, and PF algorithms, while relatively stable at the lower noise level, showed a pronounced decline as noise increased (Figure 3). Even at the lowest noise level (σ=0.05), Q learning and PF at λ=0.9 demonstrated suboptimal performance. This was evident from their lower mean cumulative rewards and higher SEM in outcomes compared to other algorithms, suggesting a lower level of robustness to even minimal environmental noise (Table 1).

#### 3.1.2. Stability of SF Learning in Reaching Goal in High-Noise Environments

**Figure 4:**
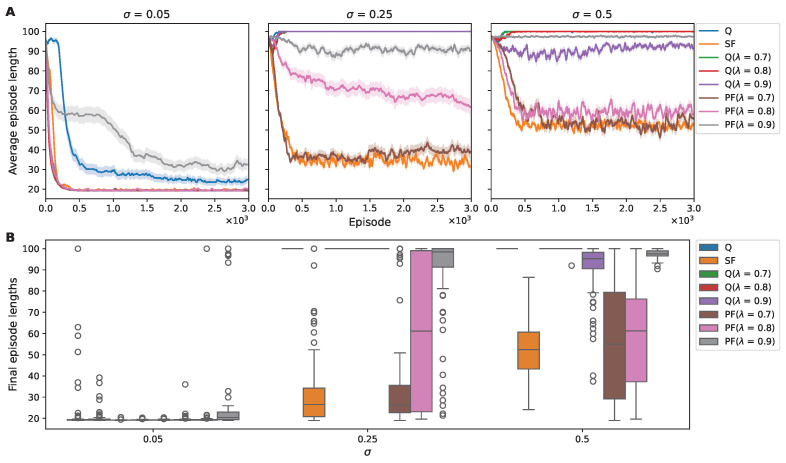
Episode length trends and distributions for RL agents in a noisy 1D environment. (**A**) The decreasing trend in average episode length over 3000 episodes for various algorithms under noise levels σ of 0.05, 0.25, and 0.5. The shaded areas represent the SEM. (**B**) The distribution of moving-averaged episode lengths in the final episodes, capturing the stabilization of learning across various noise levels. Box plots illustrate the median (central line), interquartile range (box limits), and outliers (individual points).

Our investigation into algorithmic performance begun with cumulative reward, which was subsequently followed with an examination of policy optimization efficiency, evidenced by the length of the episode steps. In low-noise conditions, most algorithms rapidly approximated the optimal behavioral policy (the left panel in Figure 4A). Q learning and PF with λ=0.9, however, exhibited a slower convergence to the optimal path. By introducing increased noise, the disparity in performance became more pronounced. Q learning and Q(λ) learning showed a notable decline in their ability to identify the optimal path consistently (Figure 4).

In contrast, the SF and PF algorithms, particularly with λ values of 0.7 and 0.8, demonstrated a robust ability to converge upon a shorter path even as noise intensified. Notably, SF maintained the smallest mean and variance in episode length across mid- and high-level noise environments (Table 2).

**Figure A1:**
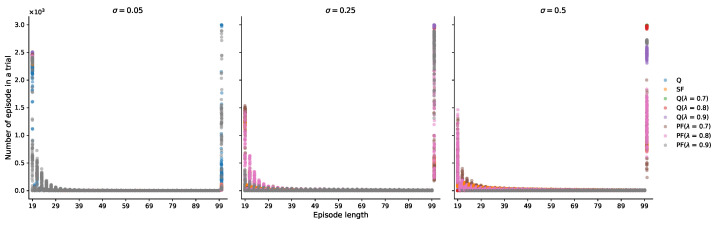
Distribution of episode lengths for RL algorithms at different noise levels in a noisy 1D environment. This figure illustrates the frequency distribution of episode lengths across 3000 trials for various RL algorithms, categorized by noise level σ = 0.05, 0.25, and 0.5. Each plot corresponds to a different noise level, showcasing the number of episodes completed within a certain number of steps. Data points represent the episode count for a given length, providing a visual comparison of algorithm efficiency and consistency.

**Figure A2:**
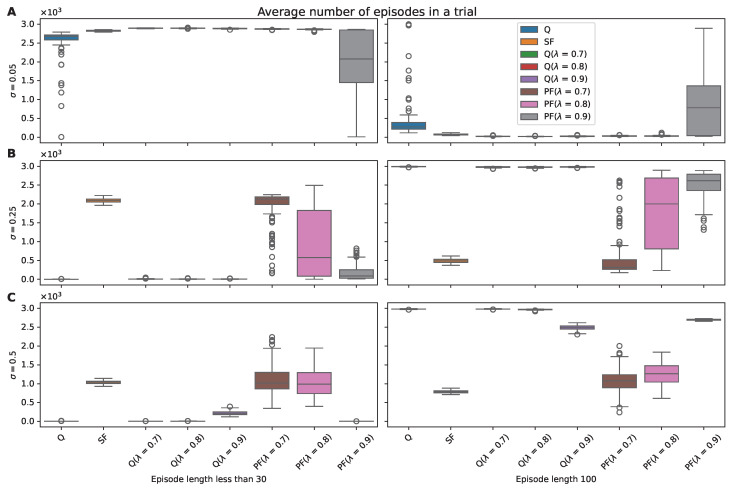
Comparison of RL algorithms for a specific range of episode lengths in a noisy 1D environment. This figure illustrates the frequency distribution of episode lengths across 3000 trials for various RL algorithms, categorized by noise levels with values of 0.05 (**A**), 0.25 (**B**), and 0.5 (**C**). The visualization concentrates on episodes ending in less than 30 steps or 100 steps. Box plots represent the distribution of the episode count for a step length, providing a visual comparison of algorithm efficiency and consistency.

We focused on the distribution of the episodes within a trial that either rapidly converged to an optimal step length or failed to do so, culminating in the maximum allowed step length of 100 (Figure A2). Our initial observation across the entire span of episode lengths indicated a rarity of episodes falling within the middle range—specifically, those in the lower 30s to the high 90s were infrequent (Figure A1). Consequently, our analysis was refined to emphasize the extremes, presenting a contrast between episodes that efficiently reached the goal and those that did not.

In the low-noise environment (σ = 0.05), the analysis revealed strong performance for most of the algorithms, with a high proportion of episodes completing in less than 30 steps, demonstrating successful convergence to optimal policies (the left panel in Figure A2A). Q learning and PF at λ=0.9 were notable exceptions, showing both diminished efficiency in achieving short episodes and an increased frequency of 100-step episodes, indicating less-reliable navigation strategies (the right panel in Figure A2A and Table A1).

A significant transformation in performance patterns emerged as noise levels increased to σ=0.25 (Figure A2B). SF maintained exceptional resilience, achieving 2084.88 ± 5.82 efficient episodes (mean ± SEM) while restricting maximum-length episodes to 493.53 ± 5.77. This markedly contrasted with the Q-learning and Q(λ) variants, which exhibited severe performance deterioration, averaging fewer than seven short episodes while experiencing a dramatic increase in maximum-length episodes (Q learning: 2990.16 ± 0.53; Q (λ=0.7): 2977.61 ± 1.23). PF with λ=0.7 maintained moderate performance with 1922.27 ± efficient episodes, though with increased performance variability.

The performance distinctions became evident under the high-noise condition (σ=0.5, Figure A2C). SF continued to show balanced performance, achieving 1037.50 ± 4.33 episodes with less than 30 steps while maintaining relatively moderate maximum-length episodes at 789.78 ± 3.86. Using PF with λ=0.7 yielded comparable robustness, resulting in efficient episodes averaging 1116.62 ± 40.41 and maximum-length episodes averaging 1072.42 ± 33.18. These results stand in sharp contrast to those of the Q-learning and Q(λ) variants, which exhibited nearly complete failure in efficient navigation (fewer than four short episodes) while predominantly resulting in maximum-length episodes (Q learning: 2981.10 ± 0.56; Q (λ=0.7): 2982.17 ± 0.46). PF with λ=0.9 demonstrated particular difficulty, achieving only 1.76 ± 0.09 efficient episodes while accumulating 2697.47 ± 1.55 maximum-length episodes.

#### 3.1.3. Policy Convergence Efficacy Under Varying Noise Conditions: A Threshold-Based Approach

**Figure 5:**
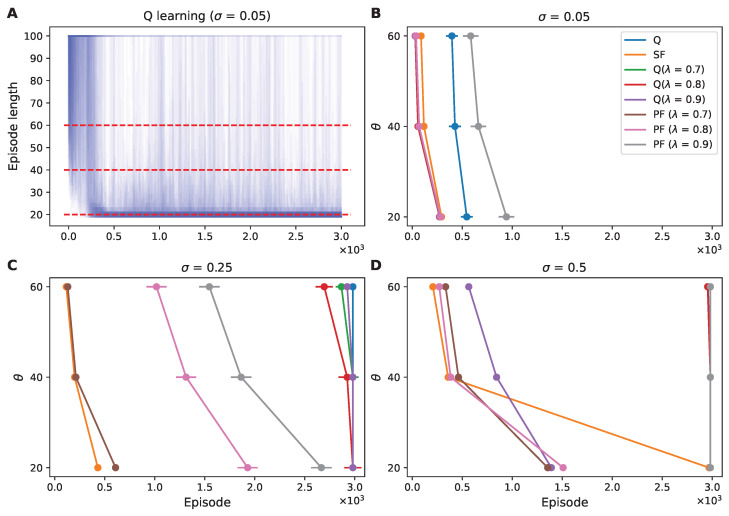
Convergence trends of RL algorithms under variable noise conditions. (**A**) Visualization of 100 episodes’ length trajectories for Q learning at a low noise level (σ = 0.05), with each trajectory represented as a line with an alpha transparency of 0.01. This allows for the visualization of the density of episode lengths of over 3000 episodes, with overlapping trajectories appearing darker. The red dotted lines represent predefined performance thresholds at 20, 40, and 60 episode lengths. (**B**–**D**) These line plots chart the convergence trajectory of the RL agents, displaying the average episode number at which each algorithm first attained a predefined performance threshold (θ) across varying noise intensities: low (**B**), medium (**C**), and high (**D**). The markers denote the mean episode number where the algorithm’s performance first met the threshold, offering a measure of its learning speed and adaptability to noise. The error bars represent the SEM.

**Figure A3:**
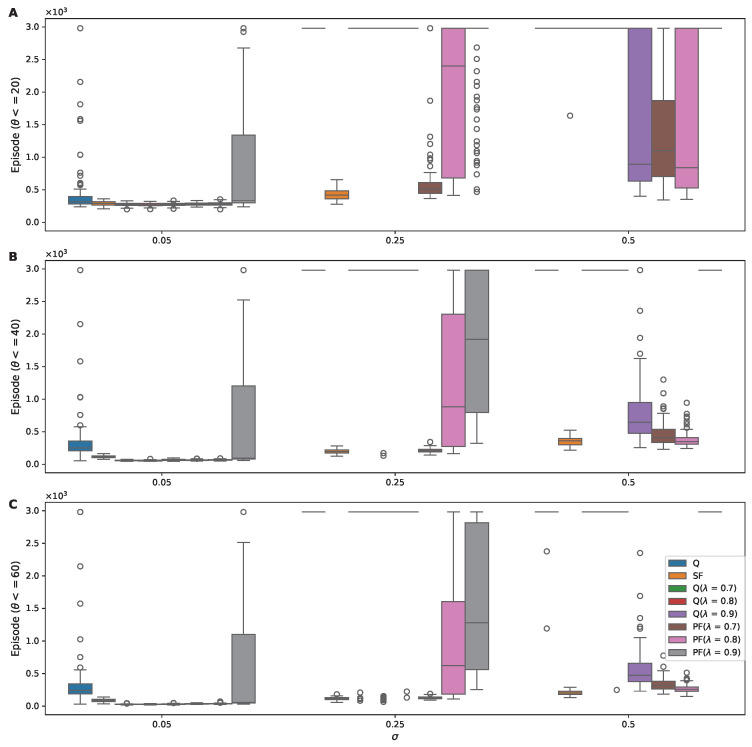
Distribution of episodes reaching 20 (**A**), 40 (**B**), 60 (**C**) steps or fewer. The box plots detail the median, interquartile range, and outliers, offering insight into each algorithm’s efficiency at each step threshold (θ) under noise level σ = 0.05, 0.25, and 0.5.

To assess how the RL agents achieved optimal convergence in the initial episodes within the 1D grid world composed of 20 states, we compared the first episode that met a specific performance threshold (θ) in each trial. Ideally, we wanted to establish a performance threshold for the RL agents to reach the goal state in 20 steps or fewer. However, as environmental noise increased, the frequency of the agents reaching this optimal number of steps decreased (Figure A2). This caused the extension of our analysis to include less-stringent thresholds, accounting for the first episodes completed within 40 and 60 steps or fewer (Figure 5 and Figure A3).

With low noise (σ = 0.05), the data showed that most algorithms, excluding-learning and PF with λ = 0.9, efficiently converged to an episode length of fewer than 20 steps within the initial few hundred episodes (Figure 5B). However, we observed a divergence in algorithmic performance as we increased the environmental noise to σ = 0.25. SF learning and PF with λ = 0.7 stood out by reaching the optimal policy in early episodes, demonstrating their robustness. In contrast, Q learning and Q(λ) learning did not exhibit this efficiency, failing to converge to the optimal policy within the 3000-episode timeframe (Figure 5C).

At the highest noise level (σ = 0.5), PF with λ = 0.7 and 0.8 as well as Q(λ) learning with λ = 0.9 maintained a commendable learning rate. SF learning, while not achieving the 20-step threshold, reached the 40- and 60-step thresholds more rapidly than the other algorithms (Figure 5D). Taken together, these results suggest that the learning efficiency and robustness of the RL algorithm to noise are nonlinear. Nevertheless, SF learning showed efficiency and robustness in reaching less-stringent step thresholds quickly at all noise levels.

### 3.2. Comparison of RL Agents in a Noisy 2D Environment

Our investigation extended into the realm of higher-dimensional noise environments to reveal if the performance trends observed in the 1D grid worlds persisted. We engaged in a series of experiments within a 2D grid world, where the agent navigated a 7 × 7 observable space, with the actual movable area being a 5 × 5 grid because of the peripheral walls (Figure 2B). The action space was expanded to four moves: up, down, left, and right. Despite the optimal path length being eight steps, we adjusted the episode length limit to 200 to account for the increased complexity arising from the larger action space and observable world size.

#### 3.2.1. Anomalous Performance Enhancements in Cumulative Reward Acquisition of RL Agents

**Figure 6:**
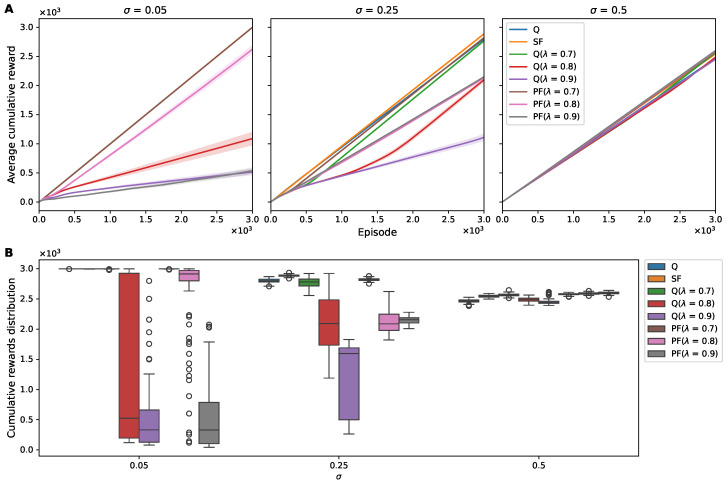
Cumulative reward of RL agents in a noisy 2D environment. (**A**) Average cumulative rewards over 3000 episodes for Q learning, Q(λ) learning, SF, and PF under different levels of observation noise (σ = 0.05, 0.25, 0.5). Each line represents the mean cumulative reward across episodes, with the shaded area depicting the SEM. (**B**) Distribution of cumulative rewards for each agent across noise settings. Box plots illustrate the median (central line), interquartile range (box limits), and outliers (individual points) for the cumulative rewards obtained over 3000 episodes, providing a comparative view of the reward distributions and the robustness of each algorithm to varying noise intensities.

We conducted a comparative analysis of the cumulative rewards achieved by different RL agents at a noise level σ of 0.05, 0.25, and 0.50, as depicted in Figure 6 and Table 3. At the low-noise level (σ = 0.05), the Q-learning, Q (λ = 0.7), SF, and PF (λ = 0.7) agents demonstrated near-optimal performance hovering close to the maximum reward, as evidenced by the negligible SEM. With a λ value of 0.8, the PF learning algorithm showed suboptimal performance with relatively a low average reward and a high SEM. In contrast, at this noise level, Q (λ = 0.8, 0.9) learning and PF (λ = 0.9) learning did not perform as well, exhibiting a lower mean reward and an elevated SEM, indicative of lesser stability in reward acquisition. As the noise level increased to σ = 0.25, a surprising uptick in accumulated rewards was noted for these latter algorithms, suggesting a possible noise threshold that might facilitate enhanced exploratory behavior or other noise-dependent dynamics not yet fully understood. SF learning, however, consistently maintained superior performance, with a relatively consistent mean/SEM ratio, suggesting a resilient performance despite the heightened noise level.

At the high noise level (σ = 0.5), an unexpected pattern emerged, where all algorithms seemed to converge toward the upper echelons of performance, a phenomenon warranting further investigation.

#### 3.2.2. Anomalous Episode Length Trends in 2D RL

Our examination of the impact of the 2D grid world’s noise on the RL algorithms extended to an in-depth analysis of episode lengths to clarify some of the unexpected trends observed in the cumulative reward outcomes.

**Figure 7:**
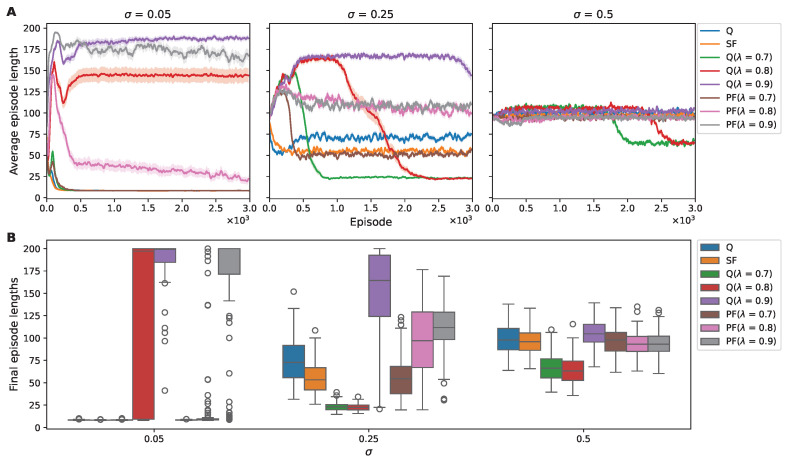
Episode length trends and distributions for RL agents in a noisy 2D environment. (**A**) The decreasing trend in average episode length across 3000 episodes for various algorithms, under a noise level σ of 0.05, 0.25, and 0.5. The shaded areas represent the SEM. (**B**) The distribution of moving-averaged episode lengths in the final episodes, capturing the stabilization of learning across various noise levels. Box plots illustrate the median (central line), interquartile range (box limits), and outliers (individual points).

In Figure 7A, we illustrate the trajectory of the average episode lengths of over 3000 episodes within a 2D noisy environment. In addition, in Figure 7B, the box plot explains the distribution of moving-averaged episode lengths in the final episodes, revealing the convergence stability exhibited by the agents. The visual representation is complemented by Table 4, which provides a statistical breakdown of the episode lengths.

At the low noise level (σ=0.05), algorithms such as Q learning, SF, Q (λ=0.7), and PF (λ=0.7) exhibited rapid alignment with the optimal path, indicating fast policy adaptation. The distribution of the moving-averaged episode lengths in the final episodes revealed that the algorithms efficiently approximated the environment’s optimal path length. The significantly low SEM values underscored the algorithms’ consistent precision, highlighting their stable performance with minimal variability.

Conversely, algorithms like PF (λ=0.8) initially produced an increase in episode lengths, hinting at initial policy inefficiencies. Nonetheless, this was mitigated over time by a decelerating trend in episode length, denoting incremental policy refinement. In the final episode, the distribution of moving-averaged episode lengths indicated that the majority, specifically the top 75%, aligned closely with the optimal path length, although there were notable outliers.

In contrast, however, Q (λ = 0.8, 0.9) learning and PF (λ = 0.9) learning displayed a less-adaptive response, with their learning curves plateauing or even worsening, suggesting a difficulty in overcoming the initial suboptimal policies.

As the noise level increased to σ = 0.25, a distinct pattern emerged for the Q-learning and SF algorithms: an initial reduction in episode lengths suggested promising policy adaptation; yet, this progress plateaued, with the algorithms failing to reach optimal levels. Algorithms employing eligibility traces diverged; they first exhibited an upsurge in average episode lengths, indicative of initial policy inefficiencies, followed by a gradual reduction. Notably, the Q (λ = 0.7, 0.8) algorithm achieved comparatively shorter episode lengths, and Q (λ = 0.8) outperformed its lower-noise-condition result. This trend is reflective of their differences in responses to increased environmental stochasticity.

At the high noise level examined (σ = 0.5), there was a general tendency for all algorithms to not significantly deteriorate or improve and to remain unchanged around a step length of 100 at the start of training. Nonetheless, Q(λ) learning, particularly at λ values of 0.7 and 0.8, demonstrated a reduction in the average episode length during the latter stages of learning. This observation suggests potential robustness to noise or an adaptive exploration strategy.

**Figure 8:**
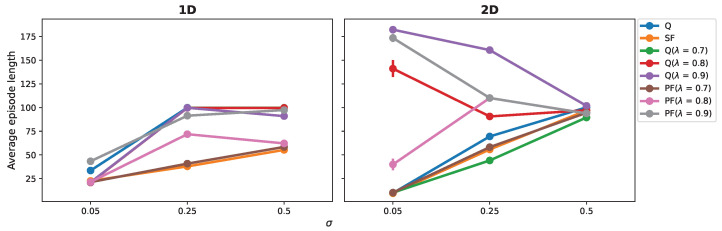
Comparative analysis of RL algorithms in noisy 1D and 2D environments. The left panel depicts the overall average episode length in the 1D grid world, while the right panel shows the same in the 2D grid world, with both averaged across 100 trials. The y axis indicates the mean episode length, representing the efficiency and consistency of learning for each algorithm. The noise levels, denoted by σ, were 0.05, 0.25, and 0.5, providing insight into each algorithm’s robustness to environmental stochasticity and their adaptability across different complexities of spatial environments.

To demonstrate the variance in the RL algorithms’ adaptation to noise across the 1D and 2D grid worlds, we analyzed the variation in the average episode lengths in response to noise level (Figure 8). Contrasting the moving-averaged episode lengths presented in the final episodes in Figure 4B and Figure 7B and Table 2 and Table 4, the average episode length was computed for each trial across total 3000 episodes and subsequently averaged across 100 trials to provide a comprehensive representation.

In the 1D domain, there was a clear trend, where most algorithms exhibited elongated average episode lengths with increasing noise level (the left panel in Figure 8). Notably, the SF algorithm demonstrated robustness, consistently keeping the average episode length shorter than that of the other algorithms.

Transitioning to the 2D grid world, the algorithms’ responses to noise became more intricate (the left panel in Figure 8). At the low noise level (σ = 0.05), the learning progress of the Q(λ)-learning and PF (λ)-learning algorithms with high λ values was hampered. However, with noise increasing to σ = 0.25, these same algorithms displayed surprising enhancements in performance. This counterintuitive phenomenon indicated a nonlinear relationship between noise levels and learning efficiency. In contrast, at the highest noise level examined (σ = 0.5), all algorithms adopted a suboptimal policy, indicating that elevated noise levels did not facilitate learning but rather resulted in a plateau in performance.

## 4. Discussion and Conclusions

The complexities of algorithmic behavior in noisy environments are highlighted by our comprehensive analysis of RL algorithms in 1D and 2D grid worlds. This section discusses the implications of our findings, the potential for algorithmic improvement, and the broader impact on the field of sensor systems.

### 4.1. Adaptation in Noisy 1D Environments

Our investigation into RL algorithm performance across 1D and 2D environments under various noise levels yielded insightful contrasts. In the 1D setting, agent performance consistently deteriorated as the noise level increased, showcasing a linear relationship between noise and efficiency (left panel in Figure 8). This trend suggests that, in simpler environments, increased noise straightforwardly hampers the agent’s ability to navigate optimally.

The robustness of the SF algorithm in the 1D environment could be attributed to its distinct approach of decomposing the value function into separate components for reward and state transition [29,30,31,33,41]. This robustness is shown in Figure 3, where SF maintains consistent performance across noise levels, with mean/SEM ratios remaining above 400 even at σ=0.25 (Table 1), while the other algorithms show marked degradation in both cumulative rewards and learning stability. In our study, only the state observations were subject to noise, leaving the reward vector unaffected. Q learning, conversely, appeared more vulnerable in noisy conditions, as it relies on direct Q-value estimation from these noisy observations [17,18]. It is important to note, however, that for effective policy making, SF learning also requires internal Q-value estimation from the reward vector and transition matrix, making it susceptible to the effects of noise, albeit to a lesser extent.

Agents employing eligibility traces, particularly PF learning with larger λ values, demonstrated a general sensitivity to noise, as evidenced by the deteriorating performance shown in Figure 3 and quantified in Table 1. For instance, at σ=0.25, PF with λ=0.9 achieved only a 498.26±35.63 cumulative reward compared to the 2508.13±5.79 for SF, representing a significant performance gap that widened further with increasing noise level. This sensitivity is further corroborated by the episode length analysis presented in Table 2, showing that PF with λ=0.9 required substantially more steps to reach the goal state under noisy conditions. This vulnerability is likely because of the way noisy observations significantly influence the state representation, affecting the computation of the trace over a longer historical window [43].

### 4.2. Adaptation in Noisy 2D Environments

The dynamics shifted when we extended our analysis to the 2D grid world. Here, we observed a departure from the linear relationship, with certain algorithms like Q learning and SF learning experiencing performance degradation as noise intensified (right panel in Figure 8). It is interesting that the Q(λ) and PF (λ) algorithms performed better at higher noise levels, particularly for a λ value of 0.9. This enhancement in performance at medium noise levels (σ = 0.25) suggested that noise-induced exploration may assist these algorithms in bypassing suboptimal local optima, leading to the development of potentially more effective policies. However, at the highest noise levels (σ = 0.5), effective policy learning seemed absent. All algorithms faced challenges in identifying optimal paths amidst high noise, indicating that the advantageous impact of noise on navigation diminishes or turns counterproductive at these intensities.

### 4.3. Optimizing λ for Noise Resilience in RL

Our comprehensive analysis of the λ parameter’s influence on algorithm performance revealed the intricate relationships between eligibility trace length and noise resilience. The data showed that λ=0.7 consistently delivered optimal performance across the environmental dimensions and noise conditions, with several key findings.

In the 1D environments, PF (λ=0.7) maintained robust performance with increasing noise level. Under medium noise (σ=0.25), it achieved 1922.27 ± 49.77 efficient episodes, while PF (λ=0.9) managed only 175.93 ± 19.79. This performance gap widened further under high noise (σ=0.5), where PF (λ=0.7) maintainsed 1116.62 ± 40.41 efficient episodes compared to just 1.76 ± 0.09 for PF (λ=0.9) (Table A1).

The impact of λ selection became even more pronounced in the 2D environments. Under low-noise conditions (σ=0.05), PF (λ=0.7) achieved a mean reward of 2997.40 compared to 537.09 for PF (λ=0.9) (Table 3) This substantial difference suggests that higher λ values, which affect a broader range of preceding states, introduce significant vulnerabilities even in relatively stable environments.

Our analysis indicates that this performance differential stems from two key mechanisms. First, higher λ values extend the temporal reach of eligibility traces, allowing noise effects to propagate across more time steps. Second, the amplification of these noise effects compounds with each affected state, creating a cascade of degraded performance. For example, at σ=0.25, PF (λ=0.9) accumulated nearly five times as many maximum-length episodes (2504.06 ± 35.52) as PF (λ=0.7) (575.55 ± 58.07, Table A1).

These findings align with those of and extend previous research Daley and Amato [44], which identified λ values between 0.6 and 0.7 as optimal for complex environments. A recent work Gupta et al. [45] on bidirectional value functions further supports the importance of effective temporal credit assignment. The approach demonstrated that these novel methods outperform traditional TD (λ) techniques in complex, noisy environments, highlighting the balance required between credit assignment depth and noise amplification.

The relationship between λ and noise resilience appeared to follow a nonlinear pattern. Performance degradation accelerated rapidly as λ increases beyond 0.7, suggesting a critical threshold where the benefits of extended temporal credit assignment were outweighed by increased noise susceptibility. This effect was evident in the transition from λ=0.8 to λ=0.9, where performance dropped precipitously across all noise conditions.

These insights emphasize the critical importance of careful λ parameter selection in practical applications. Future research should focus on developing adaptive λ selection mechanisms that can dynamically adjust to changing noise conditions, potentially allowing algorithms to maintain optimal performance across varying environmental conditions.

### 4.4. Neurobiological Foundations of RL Algorithms in Noisy Environments

In our investigation, we aligned SR learning and its derivative, SF learning, with the neurobiological mechanisms of decision making and navigation. These models encapsulate the brain’s capacity to forecast future state occupancies within spatial environments, aligning with biological plausibility [28,46]. PF learning, through its propagation of TD errors across an expanded set of preceding states, mirrors the brain’s synaptic tagging process. This process involves marking synapses for long-term memory formation [47], akin to the RL principle of eligibility traces that allocate credit to prior states based on temporal proximity.

Neuromodulatory systems like the dopamine and norepinephrine systems, which are pivotal in RL, offer a biological foundation for PF learning. Dopamine’s temporally distributed release in response to reward prediction errors hints at its role in conveying reward information across multiple prior states [48,49], while norepinephrine enhances past event representations in neural circuits, potentially aiding TD error propagation to distant states [50].

Furthermore, the integration of eligibility traces in PF learning enhances synaptic tagging, facilitating precise and resilient credit assignment. Despite evidence suggesting eligibility traces’ superiority in enhancing SF learning [34,35], our findings indicate a susceptibility to noise in PF learning at high λ values. This underscores the significance of calibrating the λ parameter amidst noise, hinting at the need for a biologically consistent decay parameter in synaptic tagging to achieve effective noise resilience.

Within cognitive map theory frameworks, SF learning adopts a forward-looking perspective, emphasizing next-state predictions, whereas PF learning retrospectively focuses on past-state history. Recent studies attribute the orbitofrontal cortex’s role in understanding prospective and retrospective continuities, with the hippocampus learning sequences using SR and PR concepts [51]. These insights affirm the importance of both perspectives in adept navigation and suggest that insights into the neural underpinnings of cognitive map learning could pave the way for more sophisticated and robust RL algorithms.

### 4.5. Limitations of This Study and Potential Future Research Directions

Our research contributes important insights into the behavior of RL algorithms within noisy environments. However, it is imperative to recognize certain limitations inherent to our study design and method, which highlight areas for further investigation.

First, while grid-world simulations are instrumental for controlled analysis, they may not capture the full spectrum of challenges inherent in real-world scenarios. This limitation could restrict the extrapolation of our results to more intricate systems and settings. However, the simplicity of grid-world simulations is beneficial, as it isolates the stochasticity arising from observational noise, allowing for a focused study on this aspect.

Second, our algorithmic comparison was narrow, concentrating on Q learning, Q(λ) learning, and the SF and PF algorithms, excluding a range of newer or alternative approaches. Nevertheless, the selection of SF and PF algorithms was motivated by their relevance and neurobiological implications. It is important to clarify that the primary aim of this research was not the development of noise-resilient algorithms but rather to understand the implications of these specific algorithms within the context of noise.

Addressing the highlighted limitations opens up several key avenues for future research:

The exploration of complex and dynamic environments represents a pivotal direction for advancing RL research. Advancing beyond simplistic grid-world simulations to study environments with dynamic features, such as moving obstacles and variable rewards, could yield deeper insights into the real-world applicability of RL algorithms [13].

Moreover, the comprehensive evaluation of RL algorithms, particularly those harnessing state-of-the-art deep learning innovations, is essential for assessing their efficiency and robustness in noisy conditions [14,15]. This would allow for a more thorough evaluation of algorithmic performance and resilience in the face of noise.

The practical application of RL algorithms, especially for tasks like robotic navigation in dynamic environments, remains an area for further investigation to determine their real-world efficacy. Conducting empirical tests of these algorithms in scenarios that mirror the unpredictability and complexity of actual conditions will be crucial for assessing their utility and adaptability [3,5,6].

Investigating the neurobiological underpinnings of SF and PF learning algorithms offers another fruitful avenue for research. By exploring their connections to hippocampal function and cognitive mapping, researchers can deepen our understanding of the biological plausibility and efficiency of these algorithms [52,53,54,55].

Leveraging insights from neurobiology to improve the efficiency of Q learning might be a potential avenue for future research. Based on the research by Grossman et al. [56], which illustrated that serotonergic neurons adjust their learning rate in response to uncertainty, adaptive seek-and-exploit strategies and dynamic learning rate modifications can enhance noise resilience.

Lastly, the significance of optimizing algorithmic parameters, such as the λ values in eligibility-trace-based algorithms, necessitates in-depth exploration. The pronounced effect of λ settings on the performance of these algorithms underscores the need for systematic studies focused on fine-tuning parameters. The adoption of automated tuning methods, aimed at calibrating algorithm configurations for enhanced efficiency in diverse environments, represents a promising direction for enhancing the robustness and applicability of RL algorithms [57].

### 4.6. Conclusions

This work offers several novel contributions to the field of RL and sensors. First, it provides empirical evidence challenging the presumed superiority of PF compared to SF in dynamic environments. Second, it elucidates the complex and nonlinear relationships between environmental noise and algorithm performance in 2D environments. Third, it provides practical insights into parameter optimization for noise robustness regarding the pivotal role of λ values in eligibility trace implementations. These findings contribute to a better understanding of RL algorithm performance regarding sensors under realistic noise conditions and provide useful guidance for implementing real-world sensor-on-board RL applications.

This investigation elucidated the intricate, nonlinear interplay between the sophistication of environmental settings and the efficacy of RL algorithms in contending with noisy conditions and executing resilient decision-making processes. Notably, the algorithms that incorporate eligibility traces exhibit varied responses to environmental noise, significantly influenced by the adjustment of the λ parameter within the 2D environment. The observed resilience of the SF algorithms, which may hint at connections to neurobiological processes, emerged as a significant insight from our study. By demonstrating the robustness of SF and PF algorithms under varying noise conditions, this study provides valuable insights for enhancing sensor-driven decision-making systems. These findings have practical implications for robotics, autonomous navigation, and sensor-based AI applications, where maintaining optimal performance despite noisy observations is critical.

## Figures and Tables

**Table 1:** Assessment of cumulative reward metrics across different noise levels in a noisy 1D environment for RL algorithms. The table summarizes the cumulative rewards for various RL agents at a noise level σ of 0.05, 0.25, and 0.5. The cumulative mean, SEM, and quartiles (25%, 50%, 75%) that map the distribution of cumulative rewards are shown. The mean/SEM ratio provides insight into the consistency of the reward relative to the average performance for each algorithm under the tested noise levels. Bold values indicate the best performance for each condition.

Noise	RL Agent	Mean	SEM	25%	50%	75%	Mean/SEM
0.05	Q	2500.85	66.05	2611.25	2724.0	2787.00	37.87
	SF	2928.07	1.80	2914.75	2931.0	2941.50	1625.43
	Q (λ = 0.7)	2978.54	0.66	2976.75	2980.0	2982.00	4479.62
	Q (λ = 0.8)	**2980.05**	0.41	2978.00	**2981.0**	2982.00	**7289.12**
	Q (λ = 0.9)	2974.02	0.82	2971.00	2975.0	2979.00	3614.60
	PF (λ = 0.7)	2968.45	0.83	2964.00	2970.0	2974.00	3579.95
	PF (λ = 0.8)	2968.10	1.37	2965.00	2971.0	2975.00	2173.90
	PF (λ = 0.9)	2186.20	83.63	1637.50	2217.0	2958.25	26.14
0.25	Q	9.96	0.53	6.00	8.0	13.00	18.91
	SF	**2508.13**	5.79	2470.25	2510.5	2553.25	**433.10**
	Q (λ = 0.7)	22.62	1.23	13.75	21.0	28.50	18.35
	Q (λ = 0.8)	25.40	1.24	17.00	25.0	31.50	20.40
	Q (λ = 0.9)	18.39	0.88	12.00	18.0	24.00	21.01
	PF (λ = 0.7)	2426.04	58.05	2481.50	**2681.5**	2736.50	41.79
	PF (λ = 0.8)	1222.49	92.20	313.75	1000.0	2195.50	13.26
	PF (λ = 0.9)	498.26	35.63	214.75	384.5	647.50	13.98
0.50	Q	19.22	0.57	15.00	18.0	22.00	33.71
	SF	**2216.88**	3.83	2193.75	**2216.0**	2248.25	**578.62**
	Q (λ = 0.7)	18.16	0.47	15.00	18.0	21.00	38.90
	Q (λ = 0.8)	32.11	1.09	25.00	30.5	35.00	29.38
	Q (λ = 0.9)	517.24	6.37	467.50	516.0	556.25	81.18
	PF (λ = 0.7)	1932.30	33.05	1768.25	1929.5	2108.00	58.47
	PF (λ = 0.8)	1760.41	27.90	1526.75	1738.5	1960.00	63.10
	PF (λ = 0.9)	309.23	1.54	298.00	308.0	321.00	201.04

**Table 2:** Distribution of moving-averaged episode length in the final episodes across different noise levels in a noisy 1D environment. The table summarizes the episode length distributions for various RL agents in the final episodes under a noise level σ of 0.05, 0.25, and 0.50. The mean episode length, SEM, and the quantiles (25%, 50%, 75%) for each algorithm are shown. Bold values indicate the best performance for each condition.

Noise	RL Agent	Mean	SEM	25%	50%	75%
0.05	Q	24.87	1.87	19.10	19.23	19.42
	SF	20.10	0.30	19.10	**19.20**	19.65
	Q (λ = 0.7)	**19.20**	0.02	19.10	**19.20**	19.21
	Q (λ = 0.8)	19.22	0.02	19.10	**19.20**	19.30
	Q (λ = 0.9)	19.22	0.02	19.10	**19.20**	19.30
	PF (λ = 0.7)	19.52	0.17	19.10	**19.20**	19.40
	PF (λ = 0.8)	20.15	0.81	19.10	19.23	19.41
	PF (λ = 0.9)	32.52	2.81	19.40	20.40	22.91
0.25	Q	100.00	0.00	100.00	100.00	100.00
	SF	**31.38**	1.57	20.79	26.52	34.27
	Q (λ = 0.7)	100.00	0.00	100.00	100.00	100.00
	Q (λ = 0.8)	100.00	0.00	100.00	100.00	100.00
	Q (λ = 0.9)	100.00	0.00	100.00	100.00	100.00
	PF (λ = 0.7)	39.14	2.81	22.68	**26.17**	35.54
	PF (λ = 0.8)	61.60	3.63	23.10	61.10	99.08
	PF (λ = 0.9)	90.13	1.92	91.31	98.50	100.00
0.50	Q	100.00	0.00	100.00	100.00	100.00
	SF	**53.13**	1.28	43.29	**52.42**	60.64
	Q (λ = 0.7)	100.00	0.00	100.00	100.00	100.00
	Q (λ = 0.8)	99.92	0.08	100.00	100.00	100.00
	Q (λ = 0.9)	91.30	1.19	90.54	95.30	98.28
	PF (λ = 0.7)	55.73	2.68	29.15	55.02	79.39
	PF (λ = 0.8)	57.90	2.36	37.25	61.25	76.25
	PF (λ = 0.9)	97.41	0.20	96.54	97.53	99.00

**Table 3:** Assessment of cumulative reward metrics across different noise levels in a noisy 2D environment for RL algorithms. The table summarizes the cumulative rewards for various RL agents with noise levels σ of 0.05, 0.25, and 0.5. The cumulative mean, SEM, and quartiles (25%, 50%, 75%) that map the distribution of cumulative rewards are shown. The mean/SEM ratio provides insight into the consistency of reward relative to the average performance of each algorithm for the tested noise levels. Bold values indicate the best performance for each condition.

Noise	RL Agent	Mean	SEM	25%	50%	75%	Mean/SEM
0.05	Q	2998.76	0.13	2998.00	**2999.00**	3000.00	23,870.59
	SF	**2998.88**	0.10	2998.00	**2999.00**	3000.00	**28,637.94**
	Q (λ = 0.7)	2997.18	0.37	2997.00	2998.00	2999.00	8008.11
	Q (λ = 0.8)	1087.93	115.40	196.75	526.50	2924.25	9.43
	Q (λ = 0.9)	514.82	54.41	128.50	332.50	660.50	9.46
	PF (λ = 0.7)	2997.40	0.30	2997.00	2998.00	2999.00	10,088.04
	PF (λ = 0.8)	2622.01	70.16	2800.25	2912.50	2970.25	37.37
	PF (λ = 0.9)	537.09	54.58	104.50	330.00	786.75	9.84
0.25	Q	2798.16	3.54	2779.75	2798.50	2826.50	789.50
	SF	**2886.03**	1.63	2877.75	**2886.00**	2896.25	**1770.18**
	Q (λ = 0.7)	2766.18	8.09	2713.25	2779.50	2828.25	342.05
	Q (λ = 0.8)	2097.67	41.30	1734.25	2094.00	2485.00	50.79
	Q (λ = 0.9)	1109.58	61.72	500.50	1595.00	1689.75	17.98
	PF (λ = 0.7)	2819.26	2.44	2805.00	2819.50	2832.25	1154.06
	PF (λ = 0.8)	2133.44	20.30	1979.75	2087.50	2246.75	105.08
	PF (λ = 0.9)	2148.33	6.01	2102.00	2154.00	2189.25	357.27
0.50	Q	2466.29	2.54	2451.00	2469.00	2482.00	971.20
	SF	2543.89	2.06	2528.75	2544.50	2560.00	1232.87
	Q (λ = 0.7)	2565.21	2.28	2551.75	2565.50	2577.50	1126.23
	Q (λ = 0.8)	2486.37	3.36	2462.75	2487.00	2513.25	739.47
	Q (λ = 0.9)	2451.33	4.73	2426.00	2440.00	2460.00	518.03
	PF (λ = 0.7)	2577.74	1.66	2569.25	2580.00	2588.00	**1554.31**
	PF (λ = 0.8)	2590.20	1.70	2579.75	2590.00	2599.25	1524.63
	PF (λ = 0.9)	**2599.42**	1.92	2585.75	**2601.50**	2613.00	1351.66

**Table 4:** Distribution of moving-averaged episode lengths in the final episodes across different noise levels in a noisy 2D environment. The table summarizes the episode length distributions for various RL agents in the final episodes at a noise level σ of 0.05, 0.25, and 0.50. The mean episode lengths, SEM, and the quantiles (25%, 50%, 75%) for each algorithm are shown. Bold values indicate the best performance for each condition.

Noise	RL Agent	Mean	SEM	25%	50%	75%
0.05	Q	8.37	0.04	8.15	8.30	8.50
	SF	**8.29**	0.03	8.10	**8.25**	8.35
	Q (λ = 0.7)	8.35	0.04	8.15	8.25	8.41
	Q (λ = 0.8)	144.24	8.25	9.19	197.22	200.00
	Q (λ = 0.9)	187.95	2.47	184.66	199.20	200.00
	PF (λ = 0.7)	8.41	0.03	8.19	8.40	8.60
	PF (λ = 0.8)	22.23	4.31	8.39	8.60	9.55
	PF (λ = 0.9)	167.44	6.16	171.31	200.00	200.00
0.25	Q	75.27	2.34	55.64	72.72	91.67
	SF	56.19	1.91	42.07	53.35	66.86
	Q (λ = 0.7)	23.28	0.48	19.81	23.00	25.71
	Q (λ = 0.8)	**22.72**	0.39	19.74	**22.52**	25.11
	Q (λ = 0.9)	144.09	5.85	124.12	164.40	192.55
	PF (λ = 0.7)	57.21	2.44	37.71	54.58	68.24
	PF (λ = 0.8)	99.14	3.92	66.99	97.08	129.10
	PF (λ = 0.9)	110.38	2.81	98.28	111.70	128.89
0.50	Q	98.90	1.73	86.83	97.80	110.67
	SF	96.05	1.41	86.27	95.85	105.65
	Q (λ = 0.7)	67.50	1.49	55.40	66.38	76.75
	Q (λ = 0.8)	**64.68**	1.50	52.65	**63.25**	74.24
	Q (λ = 0.9)	105.88	1.39	95.47	104.72	115.30
	PF (λ = 0.7)	95.96	1.48	85.41	97.93	106.31
	PF (λ = 0.8)	92.96	1.41	84.95	93.12	101.86
	PF (λ = 0.9)	93.49	1.38	85.19	93.10	102.01

## Data Availability

No datasets were used in this study. All results can be replicated using the code available at the following GitHub repository: https://github.com/HyunsuLee/PF_SF_noisy (accessed on 3 February 2025).

## Abbreviations

The following abbreviations are used in this manuscript:

RL	Reinforcement learning
SR	Successor representation
PR	Predecessor representation
SF	Successor feature
PF	Predecessor feature
MDP	Markov decision process
TD	Temporal difference
AI	Artificial intelligence
SEM	Standard error of the mean

## Appendix A

**Table A1:** Comparison of episode length distribution among algorithms and different noise conditions in a noisy 1D environment. This table presents the average number of episodes (mean ± SEM) achieved by each RL algorithm under different noise levels (σ= 0.05, 0.25, and 0.50). The analysis distinguishes between efficient navigation (less than 30 step length) and maximum-length episodes (100 step length). Bold values indicate the best performance for each condition.

Noise	RL Agent	Average Number of Episodes (Mean ± SEM)	
		Less than 30 Steps	100 Steps
0.05	Q	2540.84±41.76	499.44±66.04
	SF	2827.55±1.54	72.38±1.80
	Q (λ = 0.7)	2891.32±0.59	21.72±0.66
	Q (λ = 0.8)	2891.97±0.52	20.09±0.40
	Q (λ = 0.9)	2883.56±0.75	26.34±0.83
	PF (λ = 0.7)	2871.96±0.71	31.77±0.83
	PF (λ = 0.8)	2863.58±1.19	32.13±1.37
	PF (λ = 0.9)	2042.53±85.86	814.12±83.63
0.25	Q	2.67±0.22	2990.16±0.53
	SF	2084.88±5.82	493.53±5.77
	Q (λ = 0.7)	6.59±0.64	2977.61±1.23
	Q (λ = 0.8)	6.48±0.58	2974.85±1.23
	Q (λ = 0.9)	4.42±0.39	2981.77±0.88
	PF (λ = 0.7)	1922.27±49.77	575.55±58.07
	PF (λ = 0.8)	875.48±85.57	1778.95±92.23
	PF (λ = 0.9)	175.93±19.79	2504.06±35.52
0.50	Q	3.50±0.28	2981.10±0.56
	SF	1037.50±4.33	789.78±3.86
	Q (λ = 0.7)	2.26±0.15	2982.17±0.46
	Q (λ = 0.8)	3.76±0.22	2968.30±1.08
	Q (λ = 0.9)	221.75±5.75	2486.44±6.39
	PF (λ = 0.7)	1116.62±40.41	1072.42±33.18
	PF (λ = 0.8)	1021.22±35.98	1244.06±28.00
	PF (λ = 0.9)	1.76±0.09	2697.47±1.55
